# Feasibility and Efficacy of Online Neuropsychological Assessment

**DOI:** 10.3390/s23115160

**Published:** 2023-05-29

**Authors:** Sharon Binoy, Rachel Woody, Richard B. Ivry, William Saban

**Affiliations:** 1 Stritch School of Medicine, Loyola University, Maywood, IL 60153, USA; 2 Department of Molecular and Cell Biology, University of California, Berkeley, CA 94720, USA; 3 Center for Accessible Neuropsychology and Sagol School of Neuroscience, Tel Aviv University, Tel Aviv 69978, Israel; 4 Department of Psychology, University of California, Berkeley, CA 94720, USA; 5 Department of Occupational Therapy, Sackler Faculty of Medicine, Tel Aviv University, Tel Aviv 69978, Israel

**Keywords:** online, neuropsychological testing, Parkinson’s, ataxia, basal ganglia, cerebellum

## Abstract

Neuropsychological testing has intrinsic challenges, including the recruitment of patients and their participation in research projects. To create a method capable of collecting multiple datapoints (across domains and participants) while imposing low demands on the patients, we have developed PONT (Protocol for Online Neuropsychological Testing). Using this platform, we recruited neurotypical controls, individuals with Parkinson’s disease, and individuals with cerebellar ataxia and tested their cognitive status, motor symptoms, emotional well-being, social support, and personality traits. For each domain, we compared each group to previously published values from studies using more traditional methods. The results show that online testing using PONT is feasible, efficient, and produces results that are in line with results obtained from in-person testing. As such, we envision PONT as a promising bridge to more comprehensive, generalizable, and valid neuropsychological testing.

## 1. Introduction

Neuropsychological testing is fundamental for research, evaluation, and rehabilitation. In cognitive neuroscience, the neuropsychological assessment of patients with brain pathology has been instrumental in advancing our understanding of brain–behavior relations, with important clinical implications [1]. In addition to evaluating the cognitive changes associated with specific brain disorders, neuropsychological assessment has played a critical role in mapping particular brain regions to cognitive processes [2,3,4].

Neuropsychological tests of subcortical disorders often focus on the basal ganglia and cerebellum, with patients with Parkinson’s disease (PD) serving as a model for basal ganglia dysfunction and patients with cerebellar ataxia (CA) serving as a model for cerebellar dysfunction [5,6]. The most prominent symptoms of PD and CA are motor, with distinct impairments associated with the two disorders. In PD, the major motor symptoms [7] are typically assessed with the motor section (Part III) of the Unified Parkinson’s Disease Rating Scale (UPDRS; [8]). CA motor symptoms [9] are often assessed through the Scale for the Assessment and Rating of Ataxia (SARA; [10]).

The impact of PD and CA on cognition has been the subject of considerable debate. The Montreal Cognitive Assessment test (MoCA, [11]) is a quick, widely used tool that measures mild cognitive impairment. For both CA and PD, the literature shows mixed results from the MoCA. This may be associated with variability in symptom severity and disease progression [12,13,14].

Two other prominent domains of evaluation in the neuropsychological literature are psychiatric status and personality features. Emotional well-being can interact with cognitive and motor abilities, making it an important factor to consider [15]. The literature describes a plethora of commonly observed psychiatric disturbances in the PD population, including depression, anxiety, and apathy [16,17]. Moreover, the prevalence of anxiety and depression in individuals with severe PD is higher than in the general population of older adults [18]. Elevated rates of depression have also been reported in CA, as well as a flattened affect [19,20], something that may be anticipated given that individuals with degenerative cerebellar disorders face life-changing challenges with no known therapeutic intervention. Interestingly, anxiety does not appear to be prominent in this population [21]. Reductions in cerebellar volume have been associated with emotional dysregulation and depression [22,23,24], and cerebellar activity in healthy controls is elevated in fMRI studies of sadness and anxiety [25].

Little is known about the possible relationships between personality and these two disorders. However, pathology involving the basal ganglia and cerebellum have been linked to various personality traits. For instance, neurodegenerative disorders that affect the BG are linked to personality changes that involve negativity, restlessness, and compulsiveness [26]. In addition, a positive association has been shown between cerebellar volume and novelty seeking and a negative association with harm avoidance, two aspects of personality largely defined by actions [27]. The most common personality assessment focuses on the “Big Five” dimensions: extraversion, agreeableness, openness, conscientiousness, and neuroticism [28]. A meta-analysis of studies using instruments to measure the Big Five traits shows that PD patients score similarly to control participants in terms of neuroticism, agreeableness, and openness [29]. However, the results for conscientiousness and extraversion are more variable, with some studies reporting lower scores for PD for these dimensions [29]. To date, we are unaware of any studies examining the Big Five traits in the CA population.

Social support constitutes another factor that affects overall well-being and may be especially relevant to these populations, given that the motor symptoms of PD and CA can limit social interactions. Social support and the perception of that support became topics of particular interest during the COVID-19 pandemic when access to support groups and other sources of social support was greatly impacted [30,31]. A few studies have looked at this issue with respect to PD, with the results suggesting that perceived social support was similar in this group compared to neurotypical controls [32,33]. We did not identify any studies looking at this issue in CA.

Although there are many studies in the literature assessing the aforementioned domains of interest in PD and CA, these assessments frequently evaluate only a single patient group or domain. Within-group comparisons across many domains can produce a more complete picture at the group and individual levels. Similarly, between-group studies are relatively rare [5,21], with comparisons between PD and CA usually requiring inferences based on studies with different sampling procedures and assessment methodologies.

There are intrinsic issues in neuropsychological research that present challenges for assessing multiple domains and comparing different patient groups. Recruitment can be challenging, especially with rare neurological disorders such as CA [5]. As such, neuropsychological studies frequently have sample sizes of fewer than 15 participants [34,35,36,37,38] and the data tend to come from participants located in the same geographic area or even the same family [34,38]. This lack of diversity in neuropsychological studies may lead to a biased and unrepresentative sample. The motor impairments associated with PD and CA may also limit their ability or willingness to come to the lab for testing, and the amount of time required for thorough assessment may be challenging for these individuals.

One solution to these issues is to utilize the Internet. In the last decade, online methods have come to the forefront of behavioral research, allowing researchers to streamline the many steps of the research process, from recruitment to experimentation. Online platforms such as Amazon Mechanical Turk provide an opportunity to access large and diverse samples [39,40,41], and data from online testing have been shown to be as reliable and valid as in-person testing [40,42,43]. Online testing eliminates costs associated with transportation and remains viable during adverse events such as the COVID-19 pandemic. Overall, online assessments can greatly increase efficiency, both for researchers who can obtain more representative data and for participants who can more conveniently access research.

We developed a protocol for conducting online research, PONT (Protocol for Online Neuropsychological Testing) [5]. Using PONT, we contacted support groups to recruit a large sample of individuals with PD and CA (see Appendix A for the link to the PONT materials). Additionally, utilizing PONT, we completed ten behavioral experiments on different motor and cognitive abilities over the first three years of the pandemic, with the experiments involving over 600 participants. In the present work, we examine the feasibility and efficiency of conducting a multi-domain assessment of PD, CA, and neurotypical controls. In Experiment 1, we used standard assessments of cognition and motor function in these three groups. In Experiment 2, we conducted a more comprehensive assessment to examine depression, anxiety, perceived social support, and personality. Our primary aim is to compare our online results to published data obtained via in-person assessment within each group, rather than make comparisons between the three groups of participants. We predict that for both Experiment 1 and 2, we will obtain comparable results to values in the literature for these assessments of all three groups. 

## 2. Materials and Methods

### 2.1. Participants

Utilizing PONT, we created a large pool of participants for our online studies [5]. We recruited participants for the PONT pool via emails to support group leaders. Their email addresses were found through national support group organization websites for CA and PD. Control participants were the patients’ caregivers or recruited via advertisements posted on the Craigslist website.

### 2.2. Recruitment for Experiment 1

Drawing on the PONT pool of participants [5], email invitations were sent to 310 individuals (130 PD, 100 CA, 80 controls), inviting them to participate in a study designed to assess motor and cognitive abilities. The overall response rate to the initial email was around 25%. Follow-up emails were sent every two weeks. In total, 175 individuals responded favorably, and of those who initiated the study, the data for 3 participants (2 CA, 1 PD) were excluded because of technical problems. Thus, the final sample of participants included in the analyses reported below was composed of 172 participants: 73 PD, 60 CA, and 39 Control (see Table 1 for demographic summary). 

The CA group consisted of 26 individuals with a known genetic subtype of spinocerebellar ataxia (11 SCA3, 3 SCA2, 2 SCA1, 2 SCA5, 4 SCA6, 3 SCA28, 1 SCA42), 27 with degenerative disorders of unknown etiology, and 7 with ataxia due to other etiologies (1 ARSACS, 1 Perrault Syndrome, 1 SPG7, two autoimmune cerebellar ataxia, one gluten ataxia, 1 astrocytoma). The PD group was restricted to those who had not undergone surgical intervention as part of their treatment (e.g., DBS), and all were tested while on their current medication regimen.

### 2.3. Recruitment for Experiment 2

Email invitations were sent to approximately 148 individuals (64 PD, 24 CA, 60 Control) for the more comprehensive online assessment. The overall response rate to this first email was around 27% and, as with Experiment 1, we sent follow-up emails every 2 weeks or so, eventually resulting in a total sample of 50 individuals (16 PD, 18 CA, and 16 controls). Please see Table 2 for a complete demographic summary. The CA group was composed of 10 individuals with a known genetic subtype (SCA1 (*n* = 1); SCA2 (*n* = 3); SCA3 (*n* = 3); SCA6 (*n* = 1); SCA8 (*n* = 1); SCA42 (*n* = 1)) and eight other individuals (idiopathic ataxia (5), autoimmune cerebellar ataxia, ARSACS, gluten ataxia). One participant in the PD group did not successfully submit the PROMIS Anxiety and Depression questionnaires (*n* = 16 for ISEL-12 and IPIP-NEO-120; *n* = 15 for PROMIS Anxiety and Depression). Finally, 22 of the participants in Experiment 2 had also participated in Experiment 1 (7 Control participants, 12 CA participants, and 3 PD).

### 2.4. Online Interview: Neuropsychological Assessment and Medical Evaluation

Individuals were invited via email to participate in a live online interview. The email indicated that participation in the project required the ability to use a computer and asked the participant to indicate their preferred video platform (e.g., Zoom). In a few cases, technical problems were encountered during the initial video call, and the session was rescheduled.

The video session began with a brief explanation of the goals of the project and the completion of the informed consent forms. For both Experiment 1 and Experiment 2, the assessment protocol started with modified versions of the MoCA to evaluate the cognitive status of all participants and, for those in the PD and CA groups, a motor assessment (see below), following the procedure for online testing developed for the PONT platform (see Step 3 PONT protocol [9]). For the MoCA, we eliminated the “alternating trail-making” task since this would require that the participant have a paper copy of the task.

In both experiments, participants with PD or CA then proceeded to the medical evaluation in which they responded to questions regarding age at diagnosis, medication, other neurological or psychiatric conditions, and other relevant information (e.g., deep brain stimulation for PD, genetic subtype for CA). Following this, we assessed motor function, administering a modified version of the motor section of the UPDRS to the participants with PD and a modified version of the SARA to the participants with CA. We eliminated or modified items on the UPDRS and SARA that either require physical contact by the experimenter or might entail risk of fall. For the UPDRS, we eliminated the “postural stability task” because it requires that the experimenter pull on the shoulders of the participant. We modified three items deemed risky (“arising from chair”, “posture”, and “gait”), obtaining self-reports from the participant rather than having them perform the maneuver [44]. Similarly, we obtained self-reports of stance and gait for the SARA. For the self-reports, the participant verbally responded to a series of questions about their typical performance in each motor task and was assigned a number based on the existing SARA scale (e.g., on the SARA item for gait, from 0 = normal/no difficulty to 8 = unable to walk even when supported).

The scores for the MoCA and UPDRS batteries were adjusted to reflect these modifications. For the online MoCA, the observed score was divided by 29 (the maximum online score) and then multiplied by 30 (the maximum score on the standard test). Hence, if a participant obtained a score of 26, the adjusted score was (26/29) × 30, or 26.9. The same adjustment procedure was performed for the UPDRS. No adjustment was required for the SARA. These modifications might lead to differences in interpretation since portions of the tasks are assessed differently. However, we expect that these changes will not significantly affect the scores. A previous study that eliminated the same rigidity and postural stability tasks yielded similar relative scores to scores obtained from UPDRS assessments in which the score was based on all items [44].

The cognitive and motor questionnaires administered in Experiment 1 were given to participants tested in Experiment 2. In addition, we administered four additional online surveys to assess social support, emotional well-being (depression and anxiety), and personality traits. The Patient-Reported Outcomes Measurement Information System (PROMIS; [45]) was used to assess anxiety and depression. The PROMIS anxiety scale consists of seven statements assessing “self-reported fear, anxious misery, hyperarousal, and somatic symptoms related to arousal” and the PROMIS depression scale contains eight statements assessing “self-reported negative mood, views of self, and social cognition, as well as decreased positive affect and engagement”. The Interpersonal Support Evaluation List (ISEL-12; [46]), consisting of 12 statements was used to assess social support. The NEO International Personality Item Pool (IPIP-NEO-120; [47]) was used to assess personality. This instrument assesses the “Big Five” personality dimensions of neuroticism, extraversion, openness, agreeableness, and conscientiousness. The PROMIS and ISEL-12 questionnaires were administered via the Google Forms platform. The IPIP-NEO-120 was administered via the Qualtrics platform. To avoid any confounding order effects, we counterbalanced the order in which the four questionnaires were administered.

For Experiment 1, the interview lasted between 30 and 40 min for the control participants and 50–60 min for the PD and CA participants. For Experiment 2, the interview lasted 40–50 min for the control participants and 60–70 min for the participants with PD and CA. The materials used in our interview and evaluation are available online (Online Neuropsychological Assessment Protocol, https://docs.google.com/document/d/1e2HseTEzFERxG7OoGrd6kguFEWkVXExSgFK0WOUf0PQ/edit (accessed on 20 May 2023)). 

After completing the online interview session and questionnaires, participants were sent an email regarding their preferred payment option. Participants were reimbursed at USD 20/h, and were paid by check sent via regular mail, via PayPal, or with an Amazon gift card. Each participant was sent an automated thank-you email including a link to a short online form to provide feedback on their experience (e.g., rate difficulty, level of engagement).

### 2.5. Statistical Analysis

We performed two types of analyses. First, to compare our online data to published values in the literature, we used one-sample z-tests (when the variance is known) and *t*-tests (when the variance is unknown). As this was an exploratory study, we did not correct for multiple comparisons. Second, for comparisons between our groups (e.g., between the controls and PD), we used two-sample independent *t*-tests. We conducted chi-squared tests to compare the difference in percentage of women between groups in each experiment. We report Yates’s correction for continuity to supplement the chi-squared test results. Yates’s correction reduces the magnitude of the chi-square test to correct for the potential overestimation of statistical significance due to small sample sizes.

Whenever several suitable studies were found in the literature, we combined their results to increase the power of our proxy population statistic. For this proxy, or what we will refer to as the “literature value”, we obtained the mean and standard deviation from relevant papers and pooled these data using a weighted average calculation and Satterthwaite’s approximation for pooling standard error values to approximate population statistics. We used one-sample z-tests or *t*-tests to compare the means of our samples to these composite means (µ). Papers were selected based on their relevance (same test/questionnaire and population as the present study), with a preference for studies that administered testing in person and were published within the last 15 years.

For the MoCA, the literature value used for the control group comparison was taken from the original in-person MoCA validation study published in 2005 (*n* = 94) [11]. The composite MoCA scores for the patient groups were derived from three in-person validation studies for PD [12,13,48] and for CA [49,50,51]. For the UPDRS, we pooled three in-person studies [52,53,54]. For the SARA score, we utilized the norms published by the test’s authors [10].

For the ISEL-12, we pooled two validation studies with control groups in which the questionnaire was administered via an interview format [46,55]. We compared the PD group to a study using the ISEL-40, a longer version of the ISEL, adjusting the reported value to reflect the 12-question scale we used in the current study [46]. We did not identify papers reporting the administration of ISEL-12 to patients with CA. 

The literature value for the PROMIS tests were taken from the original norms published in 2010, based on data involving a combination of online and in-person methods [56]. For the PD group, we drew our data from a 2021 validation study comparing the PROMIS to the existing Beck Depression and Anxiety inventories [57]. Notably, the study cohort was younger on average than our cohort (µ = 62.1 vs. 72.19) and had fewer years of education (µ = 15.2 vs. 17.91). We found no prior studies employing the PROMIS scales with a CA group.

For the NEO-IPIP-120, we identified relevant papers for the control group only. These papers included the original 2014 validation study (*n* = 34,476) [52,58].

## 3. Results

We first compared the performance of our online testing for each group relative to the literature values. Given that the latter were obtained mostly in person, this comparison allows us to evaluate whether our online PONT approach produces atypical results. The average MoCA score (Table 3) for our control group was 27.8, a value that falls within the normal range on this test. We did not find a significant difference from the literature [11] (Control = 27.8 vs. µ = 27.4; z = 0.21, SD = 1.9, *p* = 0.834, effect size = 0.03). Similarly, the MoCA scores for the PD group were not significantly different from the composite, in-person mean (PD = 27.0, µ = 26.8, t = 0.63, SD = 2.4, *p* = 0.530, effect size = 0.04) [12,13,48]. However, the CA group scored significantly higher than the composite mean from in-person studies (CA = 26.0, µ = 23.6, t = 5.97, SD = 5.6, *p* < 0.001, effect size = 0.52) [49,50,51].

Comparisons between our online groups (Table S1) revealed that both patient groups performed worse than the controls on the MoCA test (PD vs. controls: t = −2.01, *p* = 0.047, effect size = 0.37; CA vs. controls: t = −3.73, *p* < 0.001, effect size = −0.73). When comparing the CA and PD groups, we found a marginally significant difference, such that the PD group has a higher score (t = 1.97, *p* = 0.051, effect size = 0.34).

For the motor assessments (Table 4), the UPDRS score (for PD) was 19.4, which was not significantly different from the literature value [52,53,54] (µ = 20.9; t = −1.36, SD = 8.7, *p* = 0.178, effect size = −0.13). Similarly, the SARA score (for CA) was 16.2, which was not significantly different from the published norm [10,51,59] (µ = 15.9; z = 0.03, SD = 8.7, *p* = 0.976, effect size = 0.04).

To assess construct validity, we performed a pair-wise comparison of the variance between the MoCA scores in Experiment 1 using Levene’s test. Similar to our comparison of the averages, we did not find significant differences in variance between the patient groups (F = 0.493, *p* = 0.484). However, the MoCA scores in each patient group were significantly different to the control group (PD vs. Control: F = 4.806, *p* = 0.031; CA vs. Control: F = 6.215, *p* = 0.014). Notably, there are differences in sample sizes between the groups (Control = 39; PD = 73; CA = 60). These unequal sample sizes can affect the accuracy and reliability of Levene’s test. To summarize, in Experiment 1, we found significant differences in variance between the patient and control groups on the MoCA, but due to the unequal sample sizes between groups, we cannot infer strong conclusions from Levene’s test about the construct validity of our analysis.

### Experiment 2

We compared the PROMIS anxiety and PROMIS depression scores from our online groups to literature values obtained from existing studies (Table 5). For our control participants, the PROMIS anxiety score of 52.0 and PROMIS depression score of 49.6 were not significantly different from published norms (Anxiety: µ = 48.5; Depression: µ = 49.3) [56]. Similarly, the online PD group had an average PROMIS anxiety score of 54.4 and PROMIS depression score of 53.1, which were not significantly different from the literature means (Anxiety µ = 50.6; Depression µ = 48.0) [33,57,60]. We could not perform a similar comparison for the CA group, given the absence of prior studies employing the PROMIS scales with CA.

In terms of between-group comparisons of the three groups in our sample (Table S2), no significant differences were observed in any of the PROMIS anxiety and PROMIS depression scores. For each test, the standard definition for clinical relevance/diagnosis is a t-score greater than 55. While our group means fell below these values, there were individuals in each group who scored above this criterion. Expressed as percentages, the values were 31.25%, 33.33%, and 46.67% for the control, CA, and PD groups, respectively, in terms of individuals showing evidence of clinically relevant depression. Comparable values for clinically relevant anxiety were 31.25%, 38.89%, and 46.67% for the control, CA, and PD groups, respectively.

Turning to the ISEL-12 test (Table 5), the control group in our sample had a mean score of 37.1, a value that is not significantly different from the literature value (µ = 39.8) [46,55]. For the PD group, the value was 38.1, also in line with the literature value for this group (ISEL-12 µ = 35.3) [32]. Our CA group average was 37.1 (no comparison group in the literature). No significant differences were found in the between-group analyses (Table S3).

The IPIP-NEO-120 test provides measures on five personality dimensions. For our control group (Table 5), the average scores were 60.6 for neuroticism, 80.4 for extraversion, 93.4 for conscientiousness, 90.3 for openness, and 94.9 for agreeableness. These values did not differ from the literature values for neuroticism (µ = 61.7), extraversion (µ = 78.9), agreeableness (µ = 93.3), and conscientiousness (µ = 93.8) [47,58]. However, there was a significant difference in the openness score, with our online sample scoring higher in this dimension compared to the literature value (µ = 83.1) [47,58]. We did not find any prior studies reporting the results from the IPIP-NEO-120 for PD and CA. Among our groups (Table S3), the control group average was significantly higher than both patient groups in the extraversion and openness dimensions. No significant differences were found between our CA and PD groups in all five personality dimensions.

In summary, where there was opportunity to make a comparison with published values, the results from our online groups yielded similar values in our measures of mental health, social support, and personality. We did not observe any differences between the PD and CA groups in any of these measures, and the only group differences were between these groups and the controls in two of the personality dimensions, with the controls showing higher levels of extraversion and openness. We note, though, that our online control sample scored higher than the literature value in the openness dimension.

To assess construct validity, we performed a pair-wise comparison of the variance of the results in every domain using Levene’s test. When looking at the PROMIS Anxiety, we did not find significant differences between any pairing of the groups (PD vs. CA: F = 0.619, *p* = 0.437; PD vs. Control: F = 0.464, *p* = 0.501; CA vs. Control: F = 0.011, *p* = 0.916). Similarly, for the PROMIS Depression, we did not find significant differences between any pairing of the groups (PD vs. CA: F = 1.276, *p* = 0.267; PD vs. Control: F = 3.318, *p* = 0.079; CA vs. Control: F = 0.493, *p* = 0.488). For the ISEL, we also did not find significant differences between any pairing of the groups (PD vs. CA: F = 0.229, *p* = 0.635; PD vs. Control: F = 0.025, *p* = 0.875; CA vs. Control: F = 0.134, *p* = 0.717). For Neuroticism, we did not find significant differences between any pairing of the groups (PD vs. CA: F = 1.370, *p* = 0.250; PD vs. Control: F = 0.829, *p* = 0.370; CA vs. Control: F = 0.085, *p* = 0.773). For Extraversion, no significant difference was found between the patient groups (PD vs. CA: F = 0.047, *p* = 0.830) and a marginally significant difference was found between the PD and control group (PD vs. Control: F = 3.986, *p* = 0.055); however, a significant difference was found between the CA and control group (CA vs. Control: F = 5.212, *p* = 0.029). For Openness, we did not find significant differences between any pairing of the groups (PD vs. CA: F = 0.158, *p* = 0.694; PD vs. Control: F = 0.017, *p* = 0.899; CA vs. Control: F = 0.090, *p* = 0.766). For Agreeableness, we did not find significant differences between any pairing of the groups (PD vs. CA: F = 1.590, *p* = 0.216; PD vs. Control: F = 0.033, *p* = 0.858; CA vs. Control: F = 0.993, *p* = 0.327). For Conscientiousness, we did not find significant differences between any pairing of the groups (PD vs. CA: F = 2.343, *p* = 0.136; PD vs. Control: F = 0.321, *p* = 0.576; CA vs. Control: F = 1.922, *p* = 0.175).

To summarize, except for one comparison between the CA and control group in Extraversion, we found no differences in variance between any pairing of the groups, supporting the construct validity of our online testing protocol.

## 4. Discussion

In this work, we utilized an online protocol, PONT, to perform neuropsychological assessments, focusing on the effect of Parkinson’s disease (PD) or cerebellar ataxia (CA) on measures of motor function, cognition, well-being, and personality. We compared the groups and, importantly, compared them to published values from studies using other traditional procedures. The latter is important for establishing the validity of online neuropsychological evaluations. Our online PONT results yielded similar results for control participants in measures of cognitive status (MoCA), anxiety and depression (PROMIS), social support (ISEL-12), and most measures of personality (IPIP-NEO-120). The only deviation from this pattern was for the measure of openness, where our control group had a higher openness score than reported in the literature. Additionally, our online assessment of motor ability (UPDRS, SARA) for the patient groups yielded similar results to papers that administer similar scales to these patient groups via in-person methods. These patterns of results support the convergent validity of our online method. Thus, the results indicate that our online method is not only feasible and efficient, but it can also produce valid results.

We also assessed the construct validity of our online method by comparing the variance of all measures between the three groups. In Experiment 1, when assessing cognition (using the MoCA), no differences in variance were found between the patient groups, but differences were found between each patient group and the control group. However, due to the unequal sample sizes between groups, one cannot infer strong conclusions from the construct validity of these analyses. In Experiment 2, except for one comparison between the CA and control group in extraversion, no differences were found in variance between any group pairings, supporting the construct validity of our online method of testing. Future studies should assess more measures of reliability and the validity of online testing.

We predicted that the motor domain would be the most difficult to conduct online given that safety concerns required that we modify some items to self-report rather than the typical clinical assessment. However, the online UPDRS and SARA scores for the PD and CA, respectively, were similar to the scores reported for these populations based on in-person testing. As mentioned above, one previous study showed that the relative ranking of impairment on the UPDRS was similar when the score was based on all items compared to when the rigidity and postural stability tasks were eliminated [44]. These are the tasks we modified or eliminated in our online version of UPDRS.

The cognitive domain was particularly interesting, given the burgeoning interest in how cognition is affected by PD and CA and what we can learn about how the basal ganglia and cerebellum support cognition in general. The online PD results on the MoCA were similar to those reported in the literature [12,13,48] and indicated a modest impairment compared to controls. The online CA group also showed an impairment relative to controls, consistent with prior findings [51]. Notably, our CA group scored significantly higher on the MoCA relative to values reported in previous studies with this population [49,50,51]. The reason for this difference is not clear. One hypothesis is that by eliminating the trail-making task, we removed a task that was more dependent on motor ability (to draw lines) than other tasks in the MoCA. This may have allowed our CA group to score better than groups in the literature.

The level of motor impairment in our CA sample was similar to the literature value, suggesting comparable levels of disease severity. We note, though, that our sample is well educated, with the average years of education indicating completion of a college degree. Although prior studies did not consistently provide this information, it would be lower given the general mean in the United States. We also note that the difference in age between our CA group and the literature CA group may have been factored into the difference in MoCA, although the association is unclear.

We also note there are significant differences in age and years of education between our online control group and the literature (Table 3). Our control group was considerably younger than control groups used in in-person studies of PD and CA. We note that MoCA scores are relatively stable among cognitively normal adults; for example, there is no age-related difference between 60–69-year-olds and 70–79-year-olds [61]. In the current study, all literature and online groups had an average age below 72.8. Therefore, age differences between cohorts should not significantly affect the MoCA score. Our control group was also better educated. However, MoCA scores are relatively stable across education levels for groups in which the years of education are above 12 [62]. Our groups and the literature groups all averaged more than 12 years of education. In sum, while there are age and education differences between our control group and the literature, we do not expect that these differences would affect performance in the MoCA. In addition, participants using the PONT protocol must have some level of computer competence, a qualification that might introduce a selection bias for those with less severe cognitive impairment.

For the PD groups, our online PONT measures of well-being produce similar results to those observed with other methods published in the literature, with mean scores elevated but not significantly so compared to controls. We could not make a direct comparison between our PONT approach and in-person PROMIS data for the CA group given that no previous studies were found. However, one in-person study utilized the Hamilton Anxiety and Depression Scale questionnaire [21]. Interestingly, the CA group in that study had significantly higher depression scores than the control group. We observed a similar pattern with the depression mean being higher in our online CA sample compared to the control group, but this difference was not significant.

We did not identify previous studies that used the IPIP-NEO-120 to assess the Big Five dimensions of personality in either PD or CA. A previous study [63] used a related questionnaire (e.g., NEO Personality Inventory) and found that PD participants show similar profiles to those observed in control participants, similar to the general pattern we find in our online PD sample. We are unaware of any literature looking at personality traits in CA. Our online CA sample had a profile similar to that observed in the PD sample.

In terms of the personality measures, our online control group had a higher openness score than the literature and also scored higher than the PD and CA groups (which had similar values). The high openness score for the control group could be due to atypical features of this sample; for example, individuals who are outwardly focused may be more likely to respond to advertisements posted on Craigslist, especially during a pandemic. Alternatively, this may have been a chance result, something that would not be unexpected given the large number of measures obtained in this study.

The results from this study highlight many of the features of online testing for neuropsychological research. In an efficient manner, we were able to assess a broad range of functional domains in cohorts of PD and CA, including domains that had not been explored in previous work (e.g., personality dimensions). Gathering comparable data sets could take years with traditional in-person methods, especially when working with a rare disorder such as CA (prevalence of 0.02%) [64]. Moreover, the online recruitment method of the PONT protocol increases the geographic diversity of the study sample.

PONT and other online approaches are well suited for neuropsychological testing because the demands on the participants are reduced. The testing can be performed at the patient’s home and, at least when automated, completed at the participant’s convenience. Participants appreciate the ease of the online format. For every session, feedback was obtained from participants regarding difficulty and level of engagement. For examples of both positive and negative feedback, see Table 2 in our previous study [5]. The convenience benefits are likely to be amplified for studies that require multiple sessions. This also reduces the economic cost to the experimenter since travel time and expenses are eliminated. Because testing is online, researchers can enlist patients who find it physically challenging to come to a lab for in-person testing.

Although there are many advantages to online testing, there are also some notable limitations to the approach in general and to our current study. First, we focused here on establishing the feasibility of performing online assessments, with validity assessed in a rather indirect manner (comparison to published values). Direct validation would require a more controlled comparison of online and in-person testing, such as administering the SARA in both methods to the same cohort. Second, it may be more difficult to standardize online testing. Each participant uses their own computer in variable settings that are not always free of distractions, factors that may be especially relevant when conducting behavioral experiments. Third, individuals who are active online would be more likely to be enlisted in online studies; this may introduce a bias against individuals with severe motor or cognitive impairment.

There are also specific limitations to the present study. First, one limitation is the difference in age between our three groups. This difference can be accounted for by the fact that there is an inherent difference in age of onset between the two patient groups—the onset of cerebellar ataxia typically occurs at an earlier age than Parkinson’s disease. Our primary aim in this study was to compare our online results to published data within each group, rather than make rigorous comparisons between the three groups of participants. Future studies can perform a more thorough assessment with larger cohorts and more specific matching of the groups in different aspects, such as age, years of education, and gender. Second, in this study we have multiple measures, which increases the chance of finding differences between cohorts when they do not exist in the population. Since this was an exploratory study, we opted to not correct for multiple comparison correction. As such, even if the few cases where we did observe significant difference either to the literature values or even between groups (e.g., in the openness measures), the results should be treated cautiously because of the increased likelihood of a Type I error.

To conclude, the current study used the PONT protocol to assay five domains in three groups. The PONT protocol is designed to implement experiments that can be conducted entirely online (self-administered or mediated through a video conferencing platform). As such, this novel approach can be employed as a tool for assessment and testing experimental hypotheses spanning a broad range of neurological disorders and domains. We envision online testing as a promising bridge to more comprehensive, generalizable, and reliable neuropsychological testing.

## Figures and Tables

**Table 1:** Experiment 1: Demographic Summary of All Groups (Mean ± SD (range)).

Group	Age (Years)	Years of Education	# of Women	Years since Diagnosis
Control (*n* = 39)	49.7 ± 11.6 (28–79)	16.1 ± 8.2 (11–20)	23	n/a
PD (*n* = 73)	66.7 ± 8.8 (47–82)	17.2 ± 2.4 (12–27)	35	7.8 ± 5.8 (0–28)
CA (*n* = 60)	59.5 ± 12.4 (29–88)	16.4 ± 2.6 (12–25)	36	14.0 ± 12.9 (0–48)

**Table 2:** Experiment 2: Demographic Summary of All Groups (Mean ± SD (range)).

Group	Age (Years)	Years of Education	# of Women	Years since Diagnosis
Control (*n* = 16)	61.3 ± 11.0 (46–78)	17.4 ± 1.4 (15–20)	11	n/a
PD (*n* = 16)	72.9 ± 16.4 (57–84)	17.9 ± 2.6 (14–25)	5	6.5 ± 5.4 (1–17)
CA (*n* = 18)	58.1 ± 11.5 (30–75)	17.1 ± 3.0 (12–25)	15	5.9 ± 4.8 (0–19)

**Table 3:** MoCA scores for patient and control groups compared to the literature. The final column is *p*-values for comparing our online control group to control literature values. Mean ± SD (range).

Feature	PD (*n* = 73)	PD Literature (*n* = 225)	CA (*n* = 60)	CA Literature (*n* = 35)	Control (*n* = 39)	Control Literature (*n* = 90)	Significance (*p*-Value)
MoCA score	27.0 ± 2.6 (19.7–30)	26.8 ± 2.4	26.0 ± 3.1 (17.6–30)	23.6 ± 5.6	27.8 ± 1.9 (23.8–30)	27.4 ± 2.0	No difference (0.834)
Age	66.7 ± 8.5 (48–82)	64.2 ± 8.8	59.5 ± 12.4 (29–88)	46.0 ± na	49.6 ± 11.9 (28–79)	72.8 ± 8.9	Significant (*p* < 0.001)
Years of Education	17.2 ± 3.1 (12–27)	13.6 ± 8.8	16.4 ± 2.3 (12–25)	Na	16.1 ± 5.6 (11–20)	13.3 ± 3.1	Significant (*p* < 0.001)

**Table 4:** UPDRS and SARA scores for motor assessment in PD and CA groups, respectively, compared to the literature (mean ± SD (range)).

Feature	PD (*n* = 73)	PD Literature *n* = 381)	CA (*n* = 60)	CA Literature (*n* = 119)
Motor	19.4 ± 6.7 (4–37)	20.9 ± 8.7	16.2 ± 7.0 (3–33)	15.9 ± 8.7
Age	66.7 ± 8.5 (47–82)	68.33 ± 35.1	59.5 ± 12.4 (29–88)	50.3 ± 13.0

No differences were found between our online data and the literature in-person values.

**Table 5:** Average scores for questionnaires assessing emotional well-being, interpersonal support, and personality traits. The final column contains *p*-values for a test comparing our online control group to control literature values. Mean ± SD (range).

Feature	PD (*n* = 16)	CA (*n* = 18)	Control (*n* = 16)	Control Literature	Significance (*p*-Value)
Anxiety	54.4 ± 10.4 (36.3–72.9)	54.0 ± 8.9 (36.3–72.9)	52.0 ± 9.2 (36.3–67.7)	48.5 ± 9.8	No difference (0.156)
Depression	53.1 ± 11.2 (37.1–68.3)	53.0 ± 9.3 (37.1–69.3)	49.6 ± 8.0 (37.1–66.4)	49.3 ± 9.6	No difference (0.864)
Interpersonal Support	38.1 ± 7.2 (27–48)	37.1 ± 6.8 (24–48)	37.1 ± 6.8 (25–46)	39.8 ± 6.7	No difference (0.146)
Neuroticism	63.3 ± 17.6 (38–96)	67.1 ± 14.0 (44–103)	60.6 ± 14 (44–86)	61.7 ± 15.7	No difference (0.755)
Extraversion	69.8 ± 14.4 (44–92)	68.0 ± 14.8 (39–90)	80.4 ± 8.8 (55–89)	78.9 ± 14.3	No difference (0.498)
Openness	83.1 ± 10.0 (66–103)	80.8 ± 13.6 (47–110)	90.3 ± 10.4 (73–108)	83.1 ± 12.6	Significant (0.01)
Agreeableness	94.9 ± 10.4 (81–110)	100.3 ± 8.1 (84–114)	94.9 ± 10.4 (74–107)	93.3 ± 11.2	No difference (0.562)
Conscientiousness	90.9 ± 16 (55–113)	94.2 ± 8.9 (74–106)	93.4 ± 12.0 (70–114)	93.8 ± 13.3	No difference (0.903)
Age	72.2 ± 9.2 (57–84)	58.1 ± 11.5 (30–75)	61.3 ± 11.2 (46–78)	n/a	n/a
Years of Education	17.9 ± 2.4 (14–25)	17.14 ± 3.0 (12–25)	17.4 ± 1.6 (15–20)	n/a	n/a

## Data Availability

The data presented in this study are available on request from the corresponding author. The data are not publicly available due to restrictions on privacy.

## Supplementary Materials

The following supporting information can be downloaded at: https://www.mdpi.com/article/10.3390/s23115160/s1, Table S1: Between-group comparisons for Experiment 1 on the MoCA; Table S2: A comparison between scores for our Experiment 2 cohorts and literature values; Table S3: Between-group comparisons on the online measures obtained in Experiment 2; Table S4: Demographic comparison for Experiment 1; Table S5: Demographic comparison for Experiment 2.

## Appendix A

Full weblink to materials for running the PONT protocol is accessible in the open science framework—https://osf.io/fktn9/ (accessed on 20 May 2023).
