# Supplementary material for: Feasibility and Efficacy of Online Neuropsychological Assessment
Source: Sensors (Basel). 2023 May 29;23(11):5160. doi: 10.3390/s23115160 (PMC10255316; doi:10.3390/s23115160)
Supplement: Supplementary file 1 [file sensors-23-05160-s001.zip › sensors-2272606-supplementary.pdf]

Supplementary Materials

Feasibility and Efficacy of Online Neuropsychological Assessment

Table S1: Between-group comparisons for Experiment 1 on the MoCA. Mean ± SD.

	Control	PD	CA		T score	T score df	P value	P Adj.	Effect Size
				**Control vs. PD**	-2.01	110	0.047	0.141	-0.37
				**Control vs. CA**	-3.73	97	<0.001	<0.001	-0.73
**MoCA**	27.8 ± 1.9	27.0 ± 2.6	26.0 ± 4.4	**CA vs. PD**	1.97	131	0.051	0.155	0.34

Table S2. Comparison between scores for our Experiment 2 cohorts and literature values.

	Cohort Mean	Literature Mean	Statistical test (Z/t)	df	P-value	Effect Size
**Anxiety**						
Control	52.0	48.5	0.36	15	0.719	0.36
PD	54.4	50.6	1.40	14	0.183	0.39
**Depression**						
Control	49.6	49.3	0.03	15	0.976	0.04
PD	53.0	48.0	1.71	14	0.109	0.51
**ISEL-12**						
Control	37.1	39.8	-0.40	15	0.689	0.39
PD	37.5	35.3	0.38	14	0.704	1.24
**Neuroticism**						
Control	60.6	61.7	-0.32	15	0.755	-0.07
**Extraversion**						
Control	80.4	78.9	0.69	15	0.498	0.13
**Openness**						
Control	90.3	83.11	3.20	15	0.006	0.72
**Agreeableness**						

Control	94.9	93.32	0.593	15	0.562	0.14
**Conscientiousness**						
Control	93.4	93.81	-0.123	15	0.903	-0.03

Table S3. Between-group comparisons for the online measures obtained in Experiment 2. Mean ± SD (range). P Adj. indicates correction for multiple comparisons.

	**Control**	**PD**	**CA**	**Comparison**	**T score**	**df**	**P-value**	**P Adj.**	**Effect Size**
**PROMIS Anxiety**	52.0 ± 9.2 (36-68)	54.4 ± 10.1 (36-73)	54.0 ± 8.9 (36-73)	**Control vs. PD**	0.68	29	0.499	1	0.24
				**Control vs. CA**	0.65	32	0.522	1	0.22
				**CA vs. PD**	-0.12	31	0.907	1	0.04
**PROMIS Depression**	49.6 ± 8.0 (37-66)	53.1 ± 10.8 (37-68)	54.0 ± 9.3 (37-69)	**Control vs. PD**	0.97	29	0.343	1	0.25
				**Control vs. CA**	1.48	32	0.149	0.792	0.51
				**CA vs. PD**	-0.11	31	0.913	1	0.04
**ISEL-12**	37.1 ± 6.8 (25-46)	37.5 ± 7.6 (27-48)	37.1 ± 6.8 (24-48)	**Control vs. PD**	0.14	29	0.893	1	0.05
				**Control vs. CA**	-0.00	32	0.995	1	-0.00
				**CA vs. PD**	-0.14	31	0.887	1	0.05
**Neuroticism**	60.6 ± 14 (44-86)	65.0 ± 17.4 (38-96)	67.1 ± 14.0 (44-103)	**Control vs. PD**	0.79	29	0.438	1	0.28
				**Control vs. CA**	1.34	32	0.190	0.630	0.46
				**CA vs. PD**	0.377	31	0.709	1	-0.13
**Extraversion**	80.4 ± 8.8 (55-89)	70.5 ± 15.2 (44-92)	68.0 ± 14.8 (39-90)	**Control vs. PD**	-2.29	29	0.032	0.080	-0.83
				**Control vs. CA**	-3.02	32	0.005	0.023	-1.02
				**CA vs. PD**	-0.48	31	0.636	1	0.17
**Openness**	90.3 ± 10.4 (73-108)	83.9 ± 10.4 (66-103)	80.78 ± 13.6 (47-110)	**Control vs. PD**	-2.05	29	0.049	0.222	-0.74
				**Control vs. CA**	-2.57	32	0.015	0.038	-0.88
				**CA vs. PD**	-0.76	31	0.435	0.984	0.26

				**Control vs. PD**	-0.02	29	0.985	1	-0.00
				**Control vs. CA**	1.66	32	0.107	0.837	0.58
**Agreeableness**	94.9 ± 10.4 (74-107)	94.8 ± 11.2 (81-110)	100.3 ± 8.1 (84-114)	**CA vs. PD**	1.60	31	0.121	0.840	-0.57
				**Control vs. PD**	-0.59	29	0.558	1	-0.21
				**Control vs. CA**	0.198	32	0.844	1	0.07
**Conscientiousness**	93.4 ± 12.0 (70-114)	90.3 ± 17.2 (55-113)	94.2 ± 8.9 (74-106)	**CA vs. PD**	0.804	31	0.431	0.738	-0.29

Table S4. Demographic comparison for Experiment 1.

	**Age**	**Education**		**%Women**
**PD vs. Control**				
**diff of means**	17.06	1.019	**chi-squared**	1.238
**SE**	2.04	0.424	**p value**	0.266
**t**	8.34	2.406	**chi-squared Yates**	0.836
**p-value**	7.53E-12	0.018	**p-value Yates**	0.361
**CD vs. Control**				
**diff of means**	9.37	0.302	**chi-squared**	0.0103
**SE**	2.507	0.459	**p value**	0.919
**t**	3.74	0.658	**chi-squared Yates**	0.012
**p-value**	<0.001	0.512	**p-value Yates**	0.914

Table S5. Demographic comparison for Experiment 2.

	Age	Education		%Women
**PD vs. Control**				
**diff of means**	11.63	0.469	**chi-squared**	4.500
**SE**	3.476	0.696	**p value**	0.034
**t**	3.34	0.67	**chi-squared Yates**	3.125
**p-value**	0.002	0.506	**p-value Yates**	0.077
**CD vs. Control**				
**diff of means**	-2.417	-0.299	**chi-squared**	1.001
**SE**	3.525	0.784	**p value**	0.317
**t**	-0.69	-0.38	**chi-squared Yates**	0.355
**p-value**	0.498	0.707	**p-value Yates**	0.551
